# The cumulative live birth rate after a freeze-only strategy versus a conventional fresh embryo transfer strategy: a call for more level 1 evidence

**DOI:** 10.1186/s12916-019-1479-2

**Published:** 2020-01-23

**Authors:** Daimin Wei, Richard S. Legro, Zi-Jiang Chen

**Affiliations:** 1 Center for Reproductive Medicine, Cheeloo College of Medicine, Shandong University, Jinan, 250001 China; 2 Department of Obstetrics and Gynecology, Penn State College of Medicine, Hershey, PA 17033 USA

**Keywords:** Freeze-only strategy, Cumulative live birth, In vitro fertilization

## Background

In recent years, much effort has been made in determining the clinical outcomes of infertility treatment. In 2013, consensus was reached that the preferred primary outcome of all infertility treatment trials is the live birth rate or cumulative live birth rate [1]. Smith et al. [2] compared a segmented versus non-segmented in vitro fertilization (IVF) cycle with the primary outcome of cumulative live birth, an important outcome to comprehensively assess the effectiveness of an IVF cycle [3]. However, the cumulative live birth rate requires a long period of follow-up until all embryos have been transferred. Furthermore, in addition to the fact that studies employ different lengths for follow-up, there are varying methods to calculate the numerator and denominator of the cumulative live birth rate [3].

As Smith et al. highlighted, frozen embryo transfer has become an integral component of IVF following the refinement of the technique for embryo cryopreservation and is being increasingly used in the clinic. Nevertheless, whether frozen embryo transfer is better than fresh embryo transfer has recently become a major topic of investigation, with several studies of varying primary outcomes being conducted.

### Study findings

Elective freezing of all embryos and the performance of a frozen embryo transfer, also known as a freeze-only strategy, has been demonstrated by randomized trials to result in a higher rate of live births in women with polycystic ovary syndrome [4] and in ovulatory women who undergo single blastocyst transfer [5] compared with a fresh embryo transfer. Nevertheless, most of the available evidence regarding the cumulative live birth rate after a freeze-only strategy compared with a fresh embryo transfer strategy is from observational studies [6, 7], including the current study by Smith et al. [2].

This study [2] represents the power of ‘big data’ to illuminate many aspects of fresh versus frozen embryo transfer, and the authors should be lauded for their thorough and exhaustive analysis of the Human Fertilisation and Embryology Authority database. The authors found that a segmented IVF cycle was associated with a 20% lower rate of cumulative live birth, compared to a non-segmented cycle, after adjusting for confounders. The decrease in the cumulative live birth rate after frozen embryo transfer was attributed to a partial reduction in the number of embryos after freezing and thawing as well as the lack of optimal regimens for endometrial preparation. Thus, their findings question the rationale of a freeze-only strategy. However, during the study period, most clinics used a slow freezing method to cryopreserve the embryos at the cleavage stage, suboptimal compared to the currently widely used method of vitrification of embryos at the blastocyst stage. Additionally, no rationale to freeze all embryos was recorded in the study, likely due to medical reasons such as high risk of ovarian hyperstimulation syndrome (OHSS), prematurely elevated progesterone, a thin endometrium, or other medical comorbidities. Thus, the scenarios assessed by Smith et al. [2] are different from the current elective freeze-only strategy, through which patients can opt for a freeze-only approach without necessarily meeting the abovementioned comorbidities. It cannot be ruled out the difference in prognosis of the study population may contribute to the difference in the rate of cumulative live birth. Additionally, results from different studies are inconsistent, with some reporting that a freeze-only strategy resulted in a similar rate of cumulative live birth compared with a fresh embryo transfer strategy [4, 5, 8].

Furthermore, as clinicians, we must frame the full view of the risk-to-benefit ratio of a treatment. If we informed a patient that segmented IVF could significantly reduce the major causes of perinatal morbidity and mortality, that is, severe prematurity (by 24%) and small-for-gestational-age (by 36%), she may accept a 20% reduction in the cumulative live birth rate since her ultimate goal is a healthy baby. More importantly, we must consider all maternal complications of the therapies to fully estimate the risk-to-benefit ratio. Smith et al. [2] found that a freeze-only strategy was associated with a higher risk of macrosomia and large-for-gestational-age as well as a lower risk of low birth weight and small-for-gestational-age babies. However, whether such an increase in birthweight is negative or positive for the long-term health of the offspring remains unknown. Additionally, a freeze-only approach has been associated with a lower risk of OHSS, but a higher risk of pre-eclampsia [4, 5]. A weakness of the study of Smith et al. [2] was that OHSS and pre-eclampsia data were not collected and thus not analyzed. We cannot make a recommendation without knowing the risks to the mother.

Thus, when the authors concluded that application of a freeze all embryos strategy “*should be restricted to those with a clinical indication*” [2], we concur it is still premature to apply a freeze-only strategy to an unselected population, but disagree with using level 2 evidence, or what the Oxford Center for Evidence-Based Medicine Levels of Evidence scale would grade as 2b evidence (equivalent to a low quality randomize controlled trial by that scale), for that conclusion. Based on level 2 evidence we would still recommend a combined hormone replacement therapy to all postmenopausal women to prevent cardiovascular disease [9]; such recommendations were ultimately refuted by level 1b evidence (i.e., a high quality randomized controlled trial) with the publication of the primary outcome of the Women’s Health Initiative [10]. In the future, as the authors also supported in their discussion, further studies, especially hypothesis-driven, high-quality randomized controlled trials, are needed to confirm the effectiveness of an elective freeze-only strategy, replicated by other similar trials, ultimately followed by their synthesis into meta-analyses forming level 1a evidence. However, since this requires years of follow-up until all embryos are used, it is difficult to design randomized trials with the cumulative live birth rate as the primary outcome unless a reasonable time period for follow-up is accepted and followed (we chose 1 year for our studies).

## Conclusions

The benefits and risks of a freeze-only strategy remain inconsistent and relatively unknown. Further level 1 studies are needed before we apply this technique to the general population.

## Data Availability

Not applicable.
